# CRISPR/Cas9-mediated generation of a tyrosine hydroxylase reporter iPSC line for live imaging and isolation of dopaminergic neurons

**DOI:** 10.1038/s41598-019-43080-2

**Published:** 2019-05-02

**Authors:** Carles Calatayud, Giulia Carola, Irene Fernández-Carasa, Marco Valtorta, Senda Jiménez-Delgado, Mònica Díaz, Jordi Soriano-Fradera, Graziella Cappelletti, Javier García-Sancho, Ángel Raya, Antonella Consiglio

**Affiliations:** 1 Department of Pathology and Experimental Therapeutics, Bellvitge University Hospital-IDIBELL, 08908 Hospitalet de Llobregat, Spain; 2 Institute of Biomedicine (IBUB) of the University of Barcelona (UB), 08028 Barcelona, Spain; 3 Center of Regenerative Medicine in Barcelona (CMRB), Hospital Duran i Reynals, Hospitalet de Llobregat, 08908 Barcelona, Spain; 4 Department of Bioscience, University of Milan, Via Festa del Perdono 7, Milan, 20122 Italy; 5 Center for Networked Biomedical Research on Bioengineering, Biomaterials and Nanomedicine (CIBER-BBN), Hospitalet de Llobregat, 08098 Barcelona, Spain; 6 Department of Condensed Matter Physics, University of Barcelona, Avinguda de la Diagonal 645, 08028 Barcelona, Spain; 7 Instituto de Biología y Genética Molecular (IBGM), Universidad de Valladolid, Calle Sanz y Forés 3, 47003 Valladolid, Spain; 8 Institució Catalana de Recerca i Estudis Avançats (ICREA), 08010 Barcelona, Spain; 9 Department of Molecular and Translational Medicine, University of Brescia, Piazza del Mercato 15, 25121 Brescia, Italy

**Keywords:** Genetic engineering, Cellular neuroscience, Parkinson's disease

## Abstract

Patient-specific induced pluripotent stem cells (iPSCs) are a powerful tool to investigate the molecular mechanisms underlying Parkinson’s disease (PD), and might provide novel platforms for systematic drug screening. Several strategies have been developed to generate iPSC-derived tyrosine hydroxylase (TH)-positive dopaminergic neurons (DAn), the clinically relevant cell type in PD; however, they often result in mixed neuronal cultures containing only a small proportion of TH-positive DAn. To overcome this limitation, we used CRISPR/Cas9-based editing to generate a human iPSC line expressing a fluorescent protein (mOrange) knocked-in at the last exon of the *TH* locus. After differentiation of the TH-mOrange reporter iPSC line, we confirmed that mOrange expression faithfully mimicked endogenous TH expression in iPSC-derived DAn. We also employed calcium imaging techniques to determine the intrinsic functional differences between dopaminergic and non-dopaminergic ventral midbrain neurons. Crucially, the brightness of mOrange allowed direct visualization of TH-expressing cells in heterogeneous cultures, and enabled us to isolate live mOrange-positive cells through fluorescence-activated cell sorting, for further differentiation. This technique, coupled to refined imaging and data processing tools, could advance the investigation of PD pathogenesis and might offer a platform to test potential new therapeutics for PD and other neurodegenerative diseases.

## Introduction

Parkinson’s disease (PD) is an incurable neurodegenerative disorder in the elderly that is characterized by the progressive loss of dopaminergic neurons (DAn) in the substantia nigra pars compacta^1^. Its incidence in Western countries is rising due to the progressive aging of the population, imposing a major burden on national healthcare systems. Despite decades of intense research, the pathogenic mechanisms of PD remain unclear owing to the lack of experimental models that recapitulate the main features of the disease. The development of induced pluripotent stem cell (iPSC) technology has opened new horizons for modeling PD, as iPSCs can be generated from patients’ somatic cells and differentiated into disease-relevant cell types, which would capture the genetic complexity of PD. Furthermore, human iPSC-derived neuronal models offer unprecedented access to the early stages of the disease, allowing the investigation of the events that initiate the pathologic process in PD^2,3^.

The development of hiPSC-based strategies to treat or model PD, however, is hampered by the lack of efficient protocols for the directed differentiation of stem cells into DAn with the appropriate characteristics of A9-subtype ventral midbrain neurons – the neuronal cell type lost in PD (for review, see^4^). Indeed, although several protocols have been developed to differentiate stem cells into functional DAn^5–8^, the differentiated progeny are a heterogeneous mix of DA and non-DA cells, which impedes identifying live DAn. Moreover, the wide variability in terms of differentiation ability among different iPSC lines represents a further limitation in the field, which hinders data interpretation^9–11^.

Several strategies have evolved in recent years to overcome these issues, based mainly on the use of DAn lineage reporters and, in particular, the expression of tyrosine hydroxylase (TH) – the first and rate-limiting step in the synthesis of dopamine – as a marker for identifying DAn^12^. However, classical approaches relying on the expression of a reporter construct controlled by the proximal promoter of the gene of interest have been unsatisfactory^13,14^. New gene-editing strategies have been more successful, enabling the insertion of reporter genes under the control of endogenous regulatory sequences. In this regard, two recent studies showed that the insertion of reporter constructs into the *TH* gene locus faithfully mimics endogenous TH expression during *in vitro* and *in vivo* differentiation^15,16^. But neither of these reporter lines allowed for the direct visualization of TH-positive (TH^+^) neurons in living cells, likely due to the low absolute expression of TH even in DAn.

In the present work, we used a CRISPR/Cas9-based editing strategy to generate a human iPSC line carrying the fluorescent protein mOrange knocked-in at the last exon of *TH*. We first confirmed the exact colocalization of mOrange expression from the reporter gene with endogenous *TH* expression in TH-mOrange hiPSC differentiated into DAn. We then used flow cytometry to evaluate mOrange reporter expression, which could be detected as early as 25 days *in vitro*. Calcium imaging experiments revealed electrophysiological differences between ventral midbrain dopaminergic neurons and non-dopaminergic neurons. This approach also enabled us to isolate pure and viable DAn cell populations from heterogeneous cultures. We thus provide a novel cellular model to study PD phenotypes *in vitro*, which may be useful for the implementation of high throughput biological discovery applications including drug target identification.

## Results

### CRISPR/Cas9-mediated knock-in of a P2A-mOrange cassette in-frame with *TH* gene

**Figure 1:**
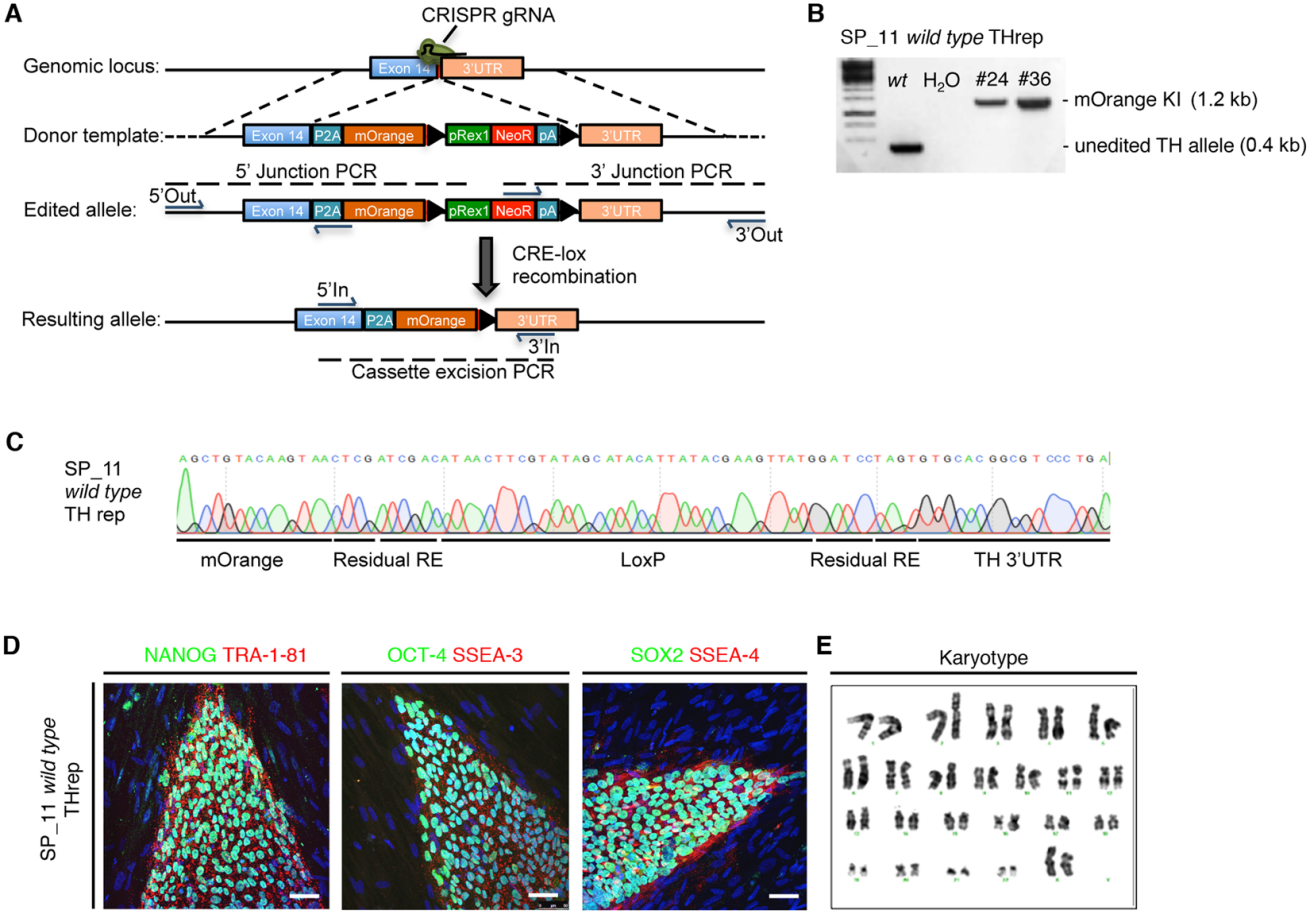
Generation of the TH-mOrange reporter iPSC line using CRISPR/Cas9-mediated gene editing. (**A**) Scheme describing the recombination steps during the edition process. Blue arrows represent the primers used for the PCR screening procedure. Black triangles represent LoxP sites surrounding the selection cassette. (**B**) Molecular analysis of the correctly targeted clones to confirm proper P2A-mOrange cassette integration and selection cassette excision in the control iPSC line. Full-length gels are included in Fig. S1C. (**C**) Sanger sequencing confirmed successful excision of the LoxP site-flanked cassette. (**D**) Immunofluorescence analysis of representative colonies of the TH reporter (SP_11) iPSC line staining positive for the pluripotency-associated markers NANOG, OCT4 and SOX2 (green) and TRA-1-81, SSEA3 and SSEA4 (red). Scale bar, 50 µm. (**E**) Normal karyotype of the TH reporter control iPSC line.

With the aim of identifying TH^+^ neurons among living cells, we generated a genetic reporter construct that could robustly and faithfully label DAn. We used CRISPR/Cas9 genome-editing to knock-in a P2A-mOrange fluorescent construct adjacent to the last exon of the *TH* gene. We chose mOrange because it is one of the brightest monomeric fluorescent proteins available^17^. The designed CRISPR/Cas9 guide RNA spacer sequence overlapped the *TH* stop codon, thereby preventing retargeting of properly edited alleles. The hiPSC line SP_11^2^, generated from a healthy control, was co-transfected with the homology-directed repair (HDR) and CRISPR/Cas9 plasmids (Fig. 1A). The HDR plasmid contained a LoxP-flanked pRex1-Neo^R^ cassette, allowing selection of recombination events. Resistant clones were molecularly characterized and the selection cassette excised (Figs 1B,C and S1). This editing approach induced >50% of biallelic integration of the exogenous sequences (data not shown). The edited hiPSC line was then expanded and characterized for the expression of pluripotency markers including OCT4, SOX2, NANOG, SSEA4, SSEA3 and TRA-1-81, and also karyotype integrity (Fig. 1D,E).

### mOrange expression faithfully recapitulates endogenous TH expression in ventral midbrain dopaminergic neurons

**Figure 2:**
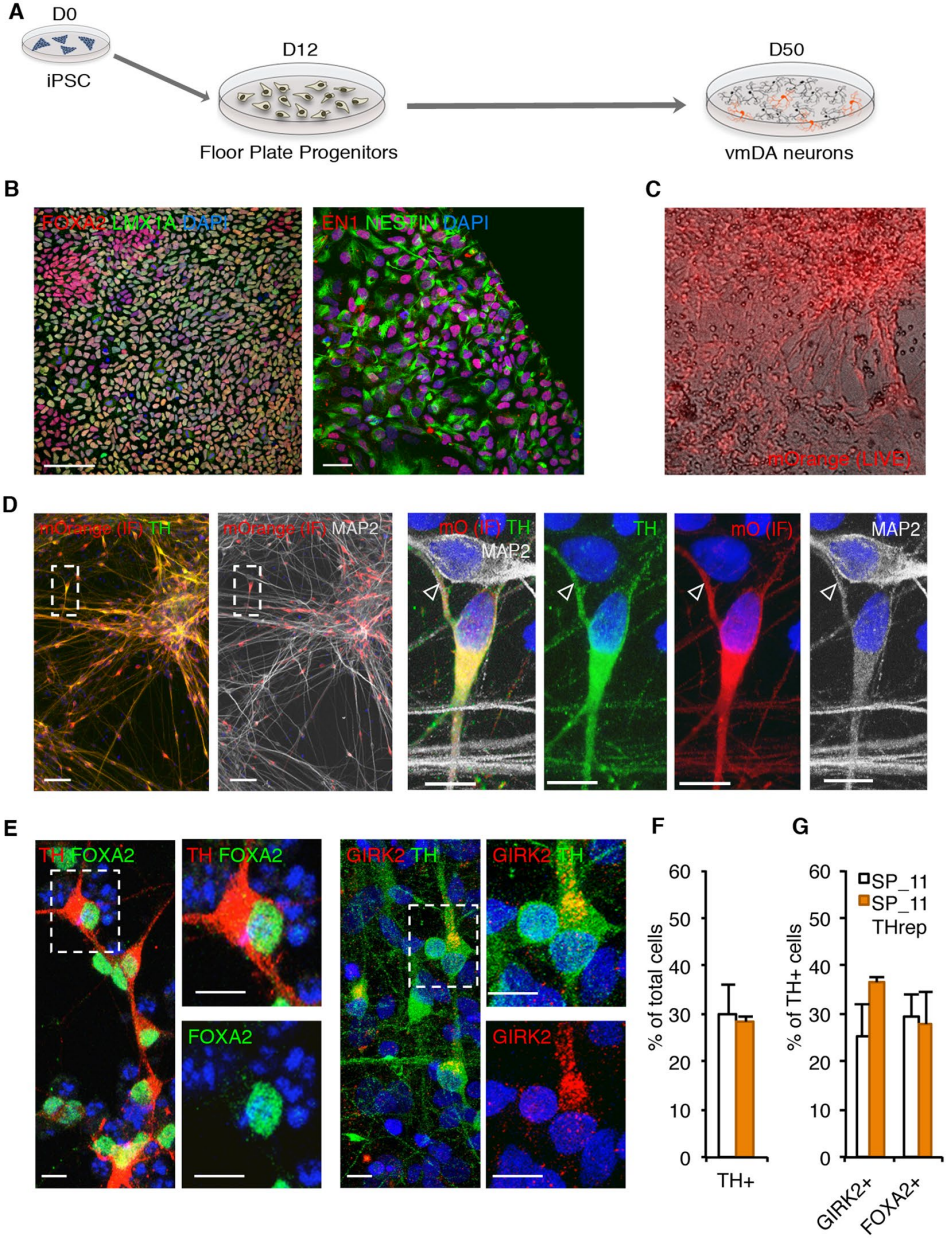
Characterization of ventral midbrain dopaminergic neurons differentiated from the TH-mOrange reporter iPSC line. (**A**) Scheme depicting iPSC differentiation towards floor plate specific ventral midbrain dopaminergic neurons (vmDAn). (**B**) Immunofluorescence analysis of representative floor plate progenitors from SP_11 TH reporter iPSCs after 13 days of ventral midbrain patterning staining positive for the floor plate markers FOXA2 and EN1 (red), LMX1A and NESTIN (green). Scale bar, 50 µm. (**C**) Live imaging of differentiated TH-mOrange iPSC cell line towards floor plate vmDAn, showing fluorescent neuronal cells after 50 days of differentiation. (**D**) Immunofluorescence analysis of DAn differentiated for 50 days and stained positive for tyrosine hydroxylase (TH; green), mOrange (red) and MAP2 (grey). Inset show in detail double-labeled mOrange^+^/TH^+^ DAn as well as double negative mOrange^−^/TH^−^ MAP2^+^ non-DAn (empty arrowhead) confirming faithful reporter activity. Nuclei were counterstained with DAPI. Scale bars 50 µm and 10 µm for the large images and the insets respectively. (**E**) Immunofluorescence analysis of DAn differentiated for 50 days and stained for TH and FOXA2 (red and green respectively; left) or GIRK2 and TH (red and green respectively; right). Nuclei were counterstained with DAPI. Scale bar, 10 µm both main image and insets. (**F**) Quantitative analysis of the percentage of cells differentiated from either original SP_11 or TH-mOrange iPSC lines stained positive for TH. (**G**) Percentage of TH+ cells stained positive for GIRK2 or FOXA2.

To differentiate the edited iPSC line towards ventral midbrain DAn (vmDAn), we followed a previously published midbrain floor plate differentiation protocol^6^ (see Methods for details) (Fig. 2A,B). Under these conditions, some fluorescent mOrange-positive cells could be observed as early as 25 days of differentiation, and the number and the fluorescence intensity of the cells increased over time. At day 50 of differentiation, cells were live-imaged (Fig. 2C) and fixed to confirm the fidelity of the reporter. Immunofluorescence analysis of fluorescent neurons revealed an absolute correlation between the mOrange signal and TH- and mRFP1-immunoreactivity (mOrange is an mRFP1 derivative) (Fig. 2D). By contrast, MAP2-positive neurons that were negative for TH immunoreactivity were also negative for mOrange (Fig. 2D), confirming the specificity of the transgene in replicating the endogenous TH expression pattern.

Further analysis at day 50 of differentiation demonstrated that ~30% of TH/mOrange-positive cells expressed the ventral midbrain forkhead box protein A2 (FOXA2) and, more importantly, the same proportion of cells also expressed the A9 domain-specific marker G protein-activated inward rectifier potassium channel 2 (GIRK2) (Fig. 2E,G). No differences between TH-mOrange edited and non-edited parental SP_11 iPSC lines were observed regarding the expression of vmDAn markers upon differentiation **(**Fig. 2F,G), indicating that differentiation was not perturbed by the insertion of the transgene.

Overall, our results show that the generated TH-mOrange iPSC line faithfully reports endogenous TH expression and that the genetic modification does not impair mDA specification.

### Ventral midbrain dopaminergic neurons have electrophysiological features distinct from non-dopaminergic neurons

**Figure 3:**
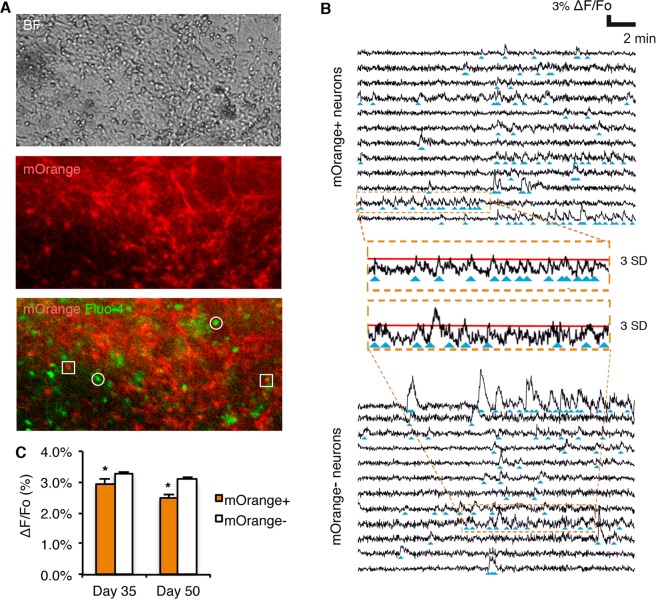
Ventral midbrain dopaminergic neurons show intrinsic electrical properties different from ventral midbrain non-dopaminergic neurons. (**A**) Live images in bright field (top picture) and the corresponding mOrange and Fluo-4 AM (middle and bottom pictures) fluorescent signal of differentiated neurons (Day 50) during calcium imaging. Squares in the bottom picture point to mOrange^+^ dopaminergic neurons whereas circles point to mOrange^−^ non-dopaminergic neurons in which oscillations of Fluo-4 AM fluorescence are measured. (**B**) Calcium traces of selected mOrange^+^ and mOrange^−^ neurons confirm electrophysiological activity. Blue arrowheads point to neuronal firing events. Insets show in detail calcium traces from individual mOrange^+^ or mOrange^−^ neurons. Red line represents an increase in fluorescence equivalent to 3 standard deviations (SD) of the mean of the baseline noise. (**C**) Quantification of the average amplitude of the firing events of mOrange^+^ and mOrange^−^ after 35 and 50 days of differentiation. Data show the average ± S.E.M. of two or three independent experiments. At day 35, 130 mOrange^+^ and 1187 mOrange^−^ cells from 3 independent experiments. At day 50, 124 mOrange^+^ and 1034 mOrange^−^ cells from 2 independent experiments. Asterisk denotes statistically significant differences (*p < 0.05).

The most defining feature of a neuron is its ability to fire action potentials in response to neurotransmitters from other neurons. In this regard, we exploited the possibility of identifying live DAn to analyze their capability to fire action potentials through studying calcium fluxes. The TH-mOrange iPSC line was differentiated towards floor plate derivatives and the resulting neuronal cultures were incubated with the calcium sensor Fluo-4 AM and imaged for periods of 30 minutes. Single calcium traces were dichotomized depending on whether they were recorded from mOrange^+^ or mOrange^−^ cells (Fig. 3A,B). Both fluorescent and non-fluorescent neurons showed sharp spontaneous increases of Fluo-4 AM signal corresponding to the sudden influxes of calcium occurring during bursts (repeated action potentials) (blue arrowheads in Fig. 3B). While both groups of neurons showed similar firing patterns, we noted small but significant differences in the amplitude of the bursts. At day 35 of differentiation, the amplitude of the burst fired by mOrange^−^ neurons was approximately 12% larger than those fired by mOrange^+^ neurons (2.954 ± 0.127 *vs* 3.296 ± 0.050; p = 0.030). This difference widened to 23% at day 50 of differentiation (2.494 ± 0.106 *vs* 3.082 +/− 0.090; p = 0.026) (Fig. 3C). These results underscore the importance of sampling a homogeneous and defined neuronal population in order to gain resolution when investigating disease-related phenotypes.

### Flow cytometry-based isolation of homogenous populations of mOrange-labeled iPSC dopaminergic neurons

**Figure 4:**
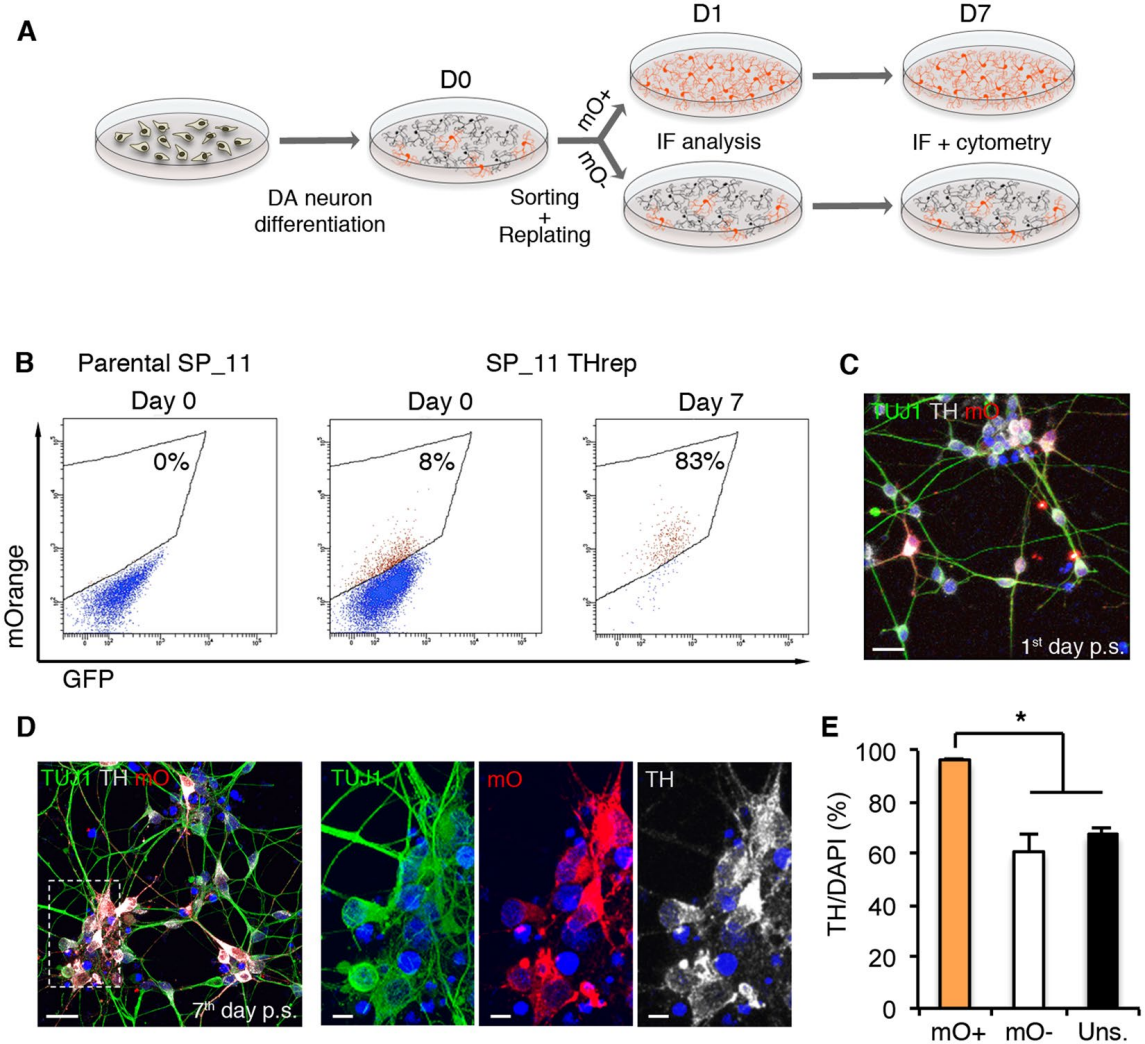
mOrange+ DA neurons are amenable to FACS-mediated purification and survive after sorting. (**A**) Experimental procedure followed for FACS-sorting and subsequent re-plating of mOrange+ DAn. (**B**) Cytograms from FACS of mOrange+ cells from differentiated TH-mOrange iPSC. Purified cells were seeded after sorting and re-analyzed after 7 days. Cytograms are representative of 2 independent experiments. (**C**,**D**) Immunofluorescence analysis of mOrange+ DAn sorted and reseeded on Matrigel for 1 (**C**) and 7 (**D**) days. One day post-sorting, living neurons were first imaged and then fixed and stained with antibodies against TUJ1 (green), TH (grey) and mOrange (red). The same combination of antibodies was used to stain neurons cultured for 7 days after sorting. Nuclei were counterstained with DAPI. Scale bars, 20 µm and 10 µm for the large image and the inset, respectively. (**E**) Quantitative analysis of the percentage of cells staining positive for TH 7 days after sorting. Data is the average ± S.E.M. of two or three independent experiments. Sorted mOrange+ cells, 1232 cells from 2 independent experiments; sorted mOrange- cells, 1020 cells from 3 independent experiments; unsorted cells, 679 cells from 2 independent experiments. Asterisk denotes statistically significant differences (*p < 0.05).

To obtain a homogenous population of mOrange-labeled iPSC-derived DAn, edited-iPSC DAn were isolated using fluorescence-activated cell sorting (FACS) at 27 days of differentiation. For this specific analysis, we utilized an alternative DAn differentiation protocol using dual-SMAD inhibition to obtain neural progenitor cells (NPCs) (Fig. S2A, B). With this protocol, NPCs can be expanded in the presence of fibroblast growth factor 2 (FGF2) and epidermal growth factor (EGF), cryopreserved, and rapidly differentiated into DAn^13,18,19^. Indeed, when we differentiated the TH-mOrange reporter line using this protocol, mOrange^+^ DAn could be visualized after only seven days of mitogen withdrawal. After 27 days of differentiation, cells were mechanically disaggregated, FACS-sorted and subsequently re-seeded on Matrigel-coated plates (Fig. 4A,B). Newly extended neurites were readily visible one day after sorting and the neuronal network continued gaining complexity over the following days (Fig. 4C,D), therefore that mOrange^+^ DAn are amenable to FACS-sorting procedures while remaining viable and maintaining their DA identity. Quantification analysis after immunostaining with an antibody to TH confirmed robust DAn purification from the mOrange^+^ sorted cells (>95% of cells were TH/mOrange-double positive). In addition, mOrange-negative sorted cells showed similar levels of mOrange^+^/TH^+^ neurons as the unsorted cells (60.8% vs. 67.5%), suggesting that the mOrange^−^ population contains mitotically active undifferentiated progenitors or neurons that have not yet switched on TH expression (Fig. 4E). These results were confirmed by FACS analysis of the sorted neurons seven days after the initial isolation **(**Fig. 4B).

Collectively, these results demonstrate that post-mitotic fluorescent DAn can be purified from heterogeneous cultures and maintained in culture after sorting.

## Discussion

Genetic reporters are extremely useful tools to study signaling and regulatory networks, and offer a potentially powerful approach to identify and specifically isolate a cell type of interest from mixed cell populations. They also allow live real-time tracking, which provides a more in-depth approach to the study cellular dynamics and fate, for example, during the development of cell therapy applications.

Here, we used CRISPR/Cas9-based gene editing to generate an iPSC reporter line carrying a copy of mOrange under control of the endogenous *TH* promoter. The mOrange fluorescent construct was preceded by a self-excisable 2A peptide sequence and was fused to the last exon of the endogenous *TH* gene. The engineered reporter system was shown to work successfully using two different DAn differentiation protocols. First, using a differentiation protocol for floor plate progenitors^6^, we demonstrated the colocalization of the reporter protein in A9 DAn, which are the most vulnerable cells during PD pathogenesis. Secondly, fluorescent DAn were obtained after differentiating TH-mOrange hiPSCs to NPCs using dual SMAD inihibtion^18^. Since earlier attempts to visualize living dopaminergic cells were unsuccessful^16^, we believe the success of our strategy is based on the exceptional brightness of the mOrange protein.

Our ability to identify live dopaminergic neurons allowed us to quantify their electrophysiological activity, an important dynamic phenotype. By calcium imaging, we could distinguish very subtle differences in firing patterns between mOrange^+^ and mOrange^−^ neurons. Specifically, we observed that mOrange^−^ neurons exhibited bursts of higher amplitude, which directly corresponds to a higher overall activity given the correlation between the fluorescence amplitude ∆F/F_0_ and the number of elicited action potentials^20^. Accordingly, the ability to sample a specific neuronal subtype should help to minimize the variability associated with the differentiation procedure. This is crucial when probing subtle and cumulative phenotypes typical of late onset disease such as PD.

Our novel genetic *TH* reporter system enabled us to successfully isolate and purifiy mOrange^+^ DAn and later resume *in vitro* culture while preserving their dopaminergic identity. Post-sorted DAn successfully attached to plates and extended new neurites in a manner similar to that observed when generating primary neuronal cultures from fetal brain. The discordance in TH^+^ neuron number estimation using cytometry or immunofluorescence is likely due to the reduced sensitivity of the former, and the absolute expression of the *TH* gene would allow for the observation of those neurons with the highest *TH* expression. However, a counterweight to this limitation is the standardization of the sampled (or isolated) neurons, since they most likely represent a defined maturation stage.

In conclusion, we here developed an efficient method for generating TH reporter iPSCs that can be differentiated and sorted to obtain pure dopaminergic neuronal populations without the commonly observed heterogeneity of iPSC-derived dopaminergic cultures. We believe that our novel TH reporter tool will facilitate future research on the processes associated with specific DAn biology and disease.

## Experimental Procedures

### Cell culture

The previously generated SP_11 iPSC line (from a healthy control subject) was used, and cells were cultured as described^2^. Briefly, hiPSC were cultured on Matrigel-coated plates (Corning, NY) and maintained in human embryonic stem cell medium consisting of KnockOut (KO)-DMEM supplemented with 20% KO-Serum Replacement (Gibco/Invitrogen, Carlsbad, CA), 2 mM ultraglutamine, non-essential amino acids and 1% penicillin/streptomycin (all from Lonza), 50 µM 2-mercaptoethanol (Gibco/Invitrogen) and 10 ng/ml bFGF (PeproTech, Rocky Hill, NJ), at 37 °C, 5% CO_2_. The medium was preconditioned overnight by irradiated mouse embryonic fibroblasts.

### Generation of CRISPR/Cas9 plasmids and donor template for homology-directed repair

The CRISPR/Cas9 plasmid pSpCas9(BB)-2A-GFP (PX458) was a gift from Dr. Feng Zhang (Broad Institute, MIT; Addgene plasmid #12345)^21^. The original pCbh promoter was exchanged for the full-length pCAGGS promoter to achieve higher expression levels in hiPSCs. Custom guide RNAs were cloned into the BbsI sites as annealed oligonucleotides. The donor template for HDR was generated using standard molecular cloning procedures. Briefly, for *TH* donor template, homology arms were amplified from genomic DNA and verified by Sanger sequencing. Resulting sequences matched those of the reference genome GRCh38. The homology arms were inserted into the KpnI-ApaI (5′HA) and SpeI-XbaI (3′HA) sites of pBS-SK (−). The sequence coding for the P2A peptide was added to mOrange with the primers used to amplify the gene and the PCR product was inserted into the ApaI-XhoI sites of the pBS-5′HA-3′HA plasmid. Finally, pRex1-Neo-SV40 was inserted between the XhoI and SpeI of the plasmid.

### Gene edition in iPSC

To generate the TH-mOrange hiPSC reporter cell line, cells were transfected with the HDR template and a Cas9- and gRNA-encoding plasmid; the latter overlapping the *TH* gene stop codon. In total, 800,000 iPSCs were seeded in 10 cm plates the day before transfection. iPSCs were co-transfected with 6 µg of CRISPR/Cas9 plasmid and 9 µg HDR template using FuGENE HD (Promega) at a 1:3 DNA to reagent ratio. Cells were plated in selection medium containing 50 µg/mL G418 (Melford Laboratories Ltd., Ipswich, UK) and maintained for 2 weeks until resistant colonies could be screened. At that time, one-half of each resistant colony was manually picked and site-specific integration was verified by PCR.

To excise the selection cassette, edited iPSCs were transfected with a CRE recombinase-expressing plasmid, gifted from Dr. Michel Sadelain (Sloan Kettering Institute; Addgene plasmid #27546)^22^. At 48 hours post-transfection, cells were dissociated and seeded at clonal density on a feeder layer of irradiated human fibroblasts. When colonies attained a certain size, they were picked and subcultured in independent Matrigel-coated wells. Cells were sampled and checked for cassette excision by PCR and Sanger sequencing. Those clones in which the cassette was excised were expanded, cryopreserved and karyotyped.

### Generation of iPSC-derived ventral midbrain dopaminergic neurons

For the derivation of vmDAn, we used a previously published protocol^6^ with minor modifications. Briefly, iPSCs were maintained in mTeSR-1 medium (StemCell Technologies, Vancouver, Canada) until they reached 80% confluence and were then cultured in serum replacement medium (day 0) (KO-DMEM, 15% KO serum, 1% non-essential amino acids, 1% GlutaMax and 1% penicillin/streptomycin) for an additional 5 days. After that, iPSCs were grown in neurobasal medium 1% N2 (17502-048; Gibco/Invitrogen), 2% B27 (17054-044; Gibco/Invitrogen) (N2B27) without vitamin A and with 1% penicillin/streptomycin. At day twelve, N2 was removed from the medium until the end of the differentiation. The medium was supplemented with SB-431542 (10 mM; day 0–day 5; Sigma-Aldrich, St Louis, MI), LDN193189 (100 nM; day 0–day 12; Miltenyi Biotec Inc., San Diego, CA), CHIR99021 (3 mM; day 3–day 25; Miltenyi), purmorphamine (2 mM; day 1–day 5; Stemgent, Cambridge, MA), Smoothened agonist (1 mM; day 1–day 5; Tocris, Bristol, UK), brain-derived neurotropic factor (BDNF) (20 ng/ml; from day 12; Miltenyi), glial cell-derived neurotropic factor (GDNF) (20 ng/ml; from day 12; Miltenyi), DAPT (10 mM; from day 12; Tocris), db-cAMP (500 mM; from day 12; Sigma-Aldrich), TGFβ3 (1 ng/ml; from day 12; Miltenyi) and ascorbic acid (AA, 200 mM; from day 12; Sigma-Aldrich). On day 20, cells were dissociated using Accutase (Merck, Kenilworth, NJ), re-plated at 0.75 × 10^5^ cells per cm^2^ on dishes pre-coated with polyornithine (15 μg/ml), laminin (1 μg/ml) and fibronectin (2 μg/ml), and cultured in neurobasal medium with 2% B27 without vitamin A and with 1% penicillin/streptomycin and trophic factors (GDNF, BDNF, TGFb3, AA, db-cAMP and DAPT) until analysis. Quantification of neurons (at day 35, 50 and 80) was assessed by confocal microscopy using anti-FOXA2, anti-MAP2, anti-TH and anti-GIRK2 antibodies.

### Generation of human neural progenitor cells

For the generation of NPCs, we followed a previously published protocol^19^. Briefly, hiPSC colonies were gently disaggregated from the culture plate and plated for 6 hours in non-adherent conditions in DMEM/F12 (Gibco/Invitrogen), 2% B27, 1% N2, 10 μM Y-27632 (Miltenyl-Biotech), 100 nM LDN-193189 (120-10 C; PeproTech), 10 μM SB431542 (S4317-5MG; Sigma-Aldrich) and 2 ng/ml bFGF. Cells were then plated for 10 days on Matrigel-coated dishes in this medium before being detached with accutase and re-plated on Matrigel-coated dishes and cultured in neural induction medium: 1:1 DMEM/F12:neurobasal medium supplemented with 2% B27, 1% N2, 1% ultraglutamine, 10 ng/ml Epidermal Growth Factor (EGF; AF-100-15 Peprotech) and 10 ng/ml bFGF. Culturing cells in this neural induction medium generates homogenous cultures of NSCs (>95% of the cells).

### NPC differentiation to dopaminergic neurons

NSCs were grown at high confluence (70%) for 7 days on Matrigel-coated dishes in N2B27 medium supplemented with 200 ng/ml Sonic Hedgehog and 100 ng/ml FGF8 (100-25; PreproTech). This first culture step was required to pattern NPCs as DAn progenitors. For terminal differentiation, DAn progenitors were plated on polyornitine/laminin-coated dishes in N2B27 medium supplemented with 20 ng/ml BDNF (450-02, PeproTech), 20 ng/ml GDNF (450-10, PeproTech), 0.5 mM db-cAMP (A6885-25MG, Sigma-Aldrich) and 5 µM DAPT (565770; Calbiochem, San Diego, CA) for the indicated time points.

### Immunofluorescence

Cells were fixed with 4% paraformaldehyde in PBS at room temperature for 15 min and permeabilized for 15 min in 0.3% Triton X-100 in TBS. Cells were then blocked in Triton X-100 with 3% donkey serum for 2 h. The following antibodies were used: goat anti-Nanog (R&D Systems, Minneapolis, MI; AF1997; 1:50), mouse IgM anti-Tra-1-81 (Merck-Millipore; MAB4381; 1:200), mouse anti-OCT4 (Santa Cruz Biotechnology, Santa Cruz, CA; sc-5279; 1:30), rat IgM anti-SSEA-3 (Developmental Studies Hybridoma Bank [DSHB] Iowa City, IA; MC-631; 1:10), mouse-SOX2 (R&D Systems; MB2018; 1:50), mouse anti-SSEA-4 (DSHB; MC-813-70; 1:100), mouse anti-NESTIN (Santa Cruz; sc-23927; 1:300action), rabbit anti-LMX1A (Millipore; ab10533; 1:1000), goat anti-EN1 (Santa Cruz; sc-46101; 1:200), mouse anti-TUJ1 (BioLegend, San Diego, CA; 801202; 1:500), chicken anti-MAP2ab (Abcam ab5392; 1:1000) goat anti-FOXA2 (R&D Systems; AF2400; 1:50), rabbit anti-TH (Santa Cruz Biotechnology; sc-14007; 1:500), sheep anti-TH (Pel-Freez Biologicals, Rogers, AR; P60101-0 1:500), rabbit anti-mRFP (Abcam, Cambridge, NA; ab34771; 1:400), rabbit anti-GIRK2 (Sigma-Aldrich; P8122; 1:40). Secondary antibodies used were the Alexa Fluor Series from Jackson ImmunoResearch Europe (Newmarket, UK) (all 1:500). Images were taken using a Leica SP5 confocal microscope. To visualize nuclei, slides were stained with 0.5 µg/ml DAPI (4′,6-diamidino-2-phenylindole) and then mounted with polyvinyl alcohol with DABCO^®^ (SIGMA).

### Calcium Imaging

At days 35 and 50 of differentiation vmDAn were incubated with the calcium indicator Fluo-4 AM (Invitrogen) for 30 minutes, with gentle shaking at room temperature. Spontaneous neuronal activity was monitored for 20 minutes with a CMOS fluorescence camera (Hamamatsu Orca Flash v4; Hamamatsu Photonics, Tokyo, Japan) at a rate of 20 images/s and a spatial resolution of 2.3 µm/pixel, which allowed for the identification of single neuronal bodies. Data acquisition was controlled through the software Hokawo 2.10 (Hamamatsu). Data analysis was carried out with the software NETCAL^23^, which registers the variation of the fluorescence signal for each neuron as a function of time, and is directly related with calcium uptake and release. The fluorescence signal for each neuron was expressed as ΔF/F_0_ (%) = 100·(F − F_0_)/F_0_, where F_0_ is the average fluorescence value of the neuron at rest.

Neuronal fluorescence traces were next analyzed with NETCAL to infer the timing of neuronal activations. The Schmitt trigger method^24^ was used for inference. This method scans the fluorescence traces for events that first pass a high threshold and then remain elevated above a second lower threshold for at least a certain minimum duration. In our analysis, we used +3 standard deviations (SD) of the mean of the baseline noise as the high threshold, +2 SD as the low threshold, and 200 ms as the minimum event length.

The series of detected activity events were further contrasted with the fluorescence signal to determine the amplitude A of each activity event. The distribution of amplitude values for mOrange^+^ and mOrange^−^ was then computed and the mean and SD values of the distributions finally evaluated.

### Statistical analysis

Differences among groups were evaluated by one-way analysis of variance, and comparisons between two groups by Student’s t-test, using Prism (Mac OS X). Error bars represent mean ± SEM. A p-value less than 0.05 was considered significant.

### Study approval

All methods were performed in accordance with the relevant guidelines and regulations. The subject from whom iPSC were obtained, gave written informed consent prior to their participation in the study. The Commission on Guarantees for Donation and Use of Human Tissues and Cells of the Instituto de Salud Carlos III (ISCIII) and the local ethics committee at the Hospital Clínic de Barcelona approved the study, in full compliance with Spanish and European laws and regulations.

## Supplementary information

Supplementary Information
