# Supplementary material for: CRISPR/Cas9-mediated generation of a tyrosine hydroxylase reporter iPSC line for live imaging and isolation of dopaminergic neurons
Source: Sci Rep. 2019 May 2;9:6811. doi: 10.1038/s41598-019-43080-2 (PMC6497635; doi:10.1038/s41598-019-43080-2)
Supplement: Supplementary file 1 — Supplementary Information [file 41598_2019_43080_MOESM1_ESM.docx]

**SUPPLEMENTARY INFORMATION**

**CRISPR/Cas9-mediated generation of a tyrosine hydroxylase reporter iPSC line for live imaging and isolation of dopaminergic neurons**

Carles Calatayud^1,2,3^, Giulia Carola^1,2^, Irene Fernández-Carasa^1,2^, Marco Valtorta^4^, Senda Jiménez^5,6^, Mònica Díaz^5,6^, Graziella Cappelletti^4^, Javier García-Sancho^7^, Jordi Soriano-Fradera^8^, Ángel Raya^5,6,9^* & Antonella Consiglio^1,2,10^*

1. Department of Pathology and Experimental Therapeutics, Bellvitge University Hospital-IDIBELL, 08908 Hospitalet de Llobregat, Spain.
2. Institute of Biomedicine (IBUB) of the University of Barcelona (UB), 08028 Barcelona, Spain.
3. Center of Regenerative Medicine in Barcelona (CMRB), Hospital Duran i Reynals, Hospitalet de Llobregat, 08908 Barcelona, Spain.
4. Department of Bioscience, University of Milan, Via Festa del Perdono 7, Milan 20122, Italy.
5. Center of Regenerative Medicine in Barcelona (CMRB), Hospital Duran i Reynals, Hospitalet de Llobregat, 08908 Barcelona, Spain
6. Centre for Networked Biomedical Research on Neurodegenerative Diseases (CIBERNED), Calle Nicolás Cabrera 1, Campus de Cantoblanco, 28049 Madrid, Spain.
7. Instituto de Biología y Genética Molecular (IBGM), Universidad de Valladolid, Calle Sanz y Forés 3, 47003 Valladolid, Spain
8. Department of Condensed Matter Physics, University of Barcelona, Avinguda de la Diagonal 645, 08028 Barcelona, Spain.
9. Institució Catalana de Recerca i Estudis Avançats (ICREA), 08010 Barcelona, Spain
10. Department of Molecular and Translational Medicine, University of Brescia, Piazza del Mercato 15, 25121 Brescia, Italy

* Correspondence should be addressed to:

consiglio@ub.edu (A.C.) or araya@cmrb.eu (A.R.)

**INDEX OF CONTENTS**

**Supplementary figures:**

**Figure S1: Screening gene edited clones**

**Figure S2: iPSC-derived neural progenitor characterization.**

**
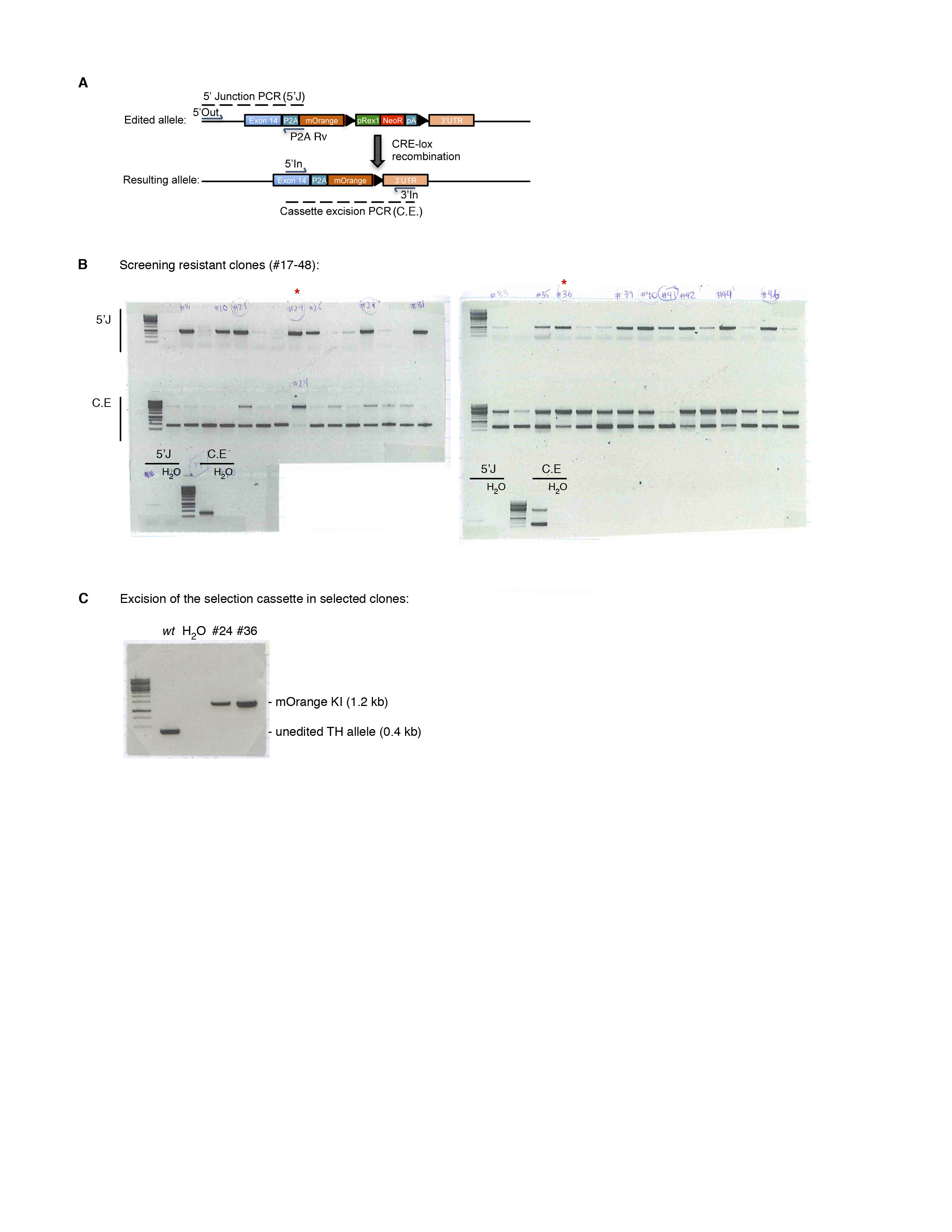
**

**Figure S1: Screening gene-edited clones.** (A) Scheme describing the different set of primers employed for checking site-specific integration of the exogenous sequences. (B) PCR-based screening of G418-resistant clones. 5’ Junction (5’J) PCR informs about locus-specific integration of the exogenous sequences since 5’Out primer anneals sequences outside the homology arms. Expected PCR product is ∼1 kb-long. Cassette Excision (C.E.) PCR informs about the zigosity of the edition. Expected product for a targeted allele is ∼3.3 kb-long whereas for the non-targeted allele is ∼0.4 kb-long. In most cases, the amplification of the non-edited allele results from G418-sensitive cells remaining within resistant colonies or to genomic DNA released by dead cells. (C) Uncropped agarose gel corresponding to Fig. 1B. PCR-based verification of selection cassette excision after CRE recombinase transfection. C.E. primers were used.

**
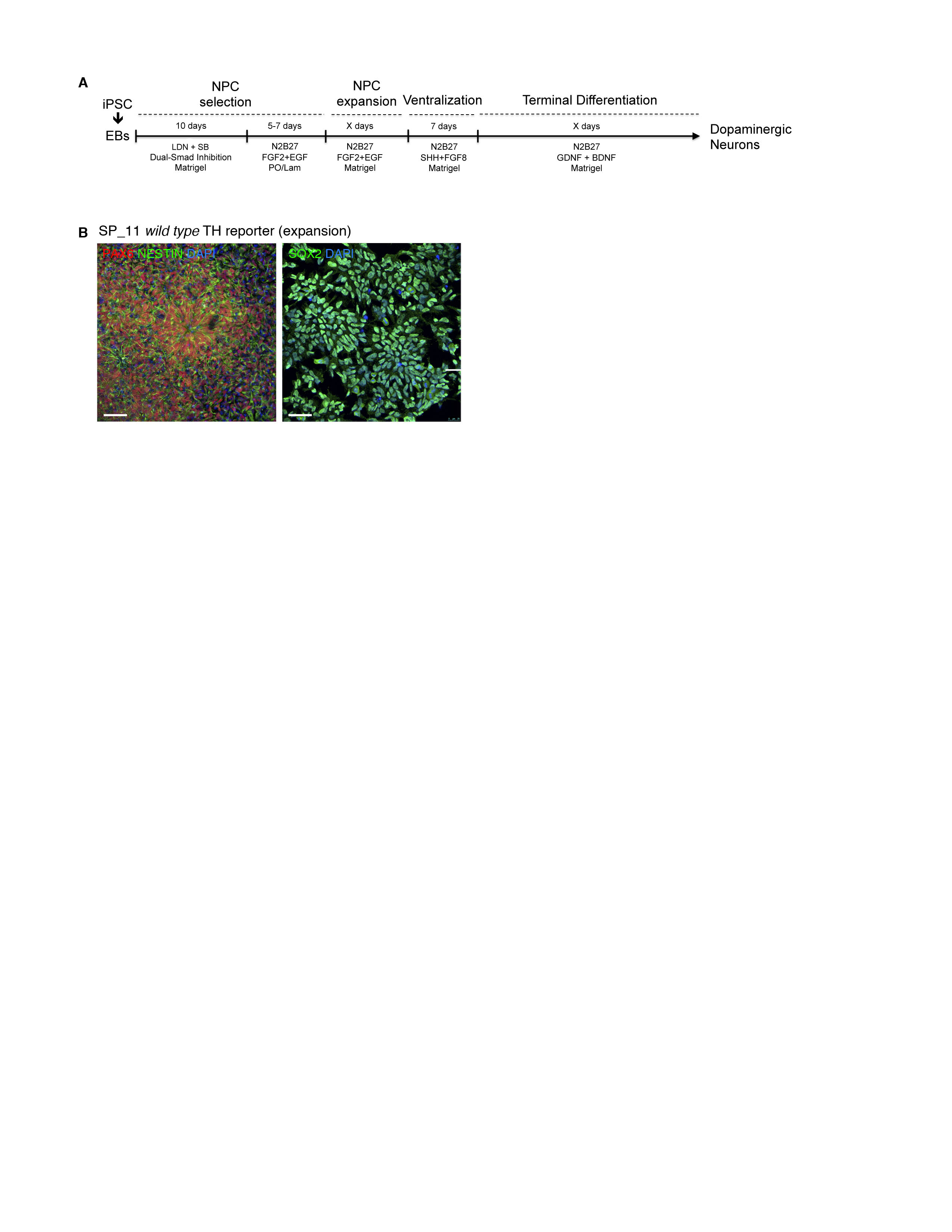
**

**Figure S2: iPSC-derived neural progenitor cells (NPCs) characterization.** (A) Scheme describing the stepwise differentiation procedure from iPSC towards neuroectoderm first, and from NPCs towards dopaminergic neurons. (B) Immunofluorescence analysis of representative neuroectodermal progenitors from TH reporter lines in expansion culture conditions stained positive for the early neuroectodermal markers PAX6 (red), NESTIN and SOX2 (green). Scale bar, 50 µm.
